# Gingival Margin Damage During Supragingival Dental Polishing by Inexperienced Operator—Pilot Study

**DOI:** 10.3390/jfb15120374

**Published:** 2024-12-11

**Authors:** Blagovesta Yaneva, Petar Shentov, Dimitar Bogoev, Maria Mutafchieva, Stela Atanasova-Vladimirova, Kiril Dimitrov, Diyana Vladova

**Affiliations:** 1 Department of Periodontology and Oral Mucosa Diseases, Faculty of Dental Medicine, Medical University of Plovdiv, 4002 Plovdiv, Bulgaria; petar.shentov@mu-plovdiv.bg (P.S.); dimitar.bogoev@mu-plovdiv.bg (D.B.); mariya.mutafchieva@mu-plovdiv.bg (M.M.); 2 Institute of Physical Chemistry “ Rostislav Kaishev”, Bulgarian Academy of Sciences, 1000 Sofia, Bulgaria; statanasova@ipc.bas.bg; 3 Department of General and Clinical Pathology, Faculty of Veterinary Medicine, Trakia University, 6000 Stara Zagora, Bulgaria; kiril.dimitrov@trakia-uni.bg; 4 Department of Veterinary Anatomy, Histology and Embryology, Faculty of Veterinary Medicine, Trakia University, 6000 Stara Zagora, Bulgaria; diyana.vladova@trakia-uni.bg

**Keywords:** air polishing, histology, gingiva, erythritol, calcium carbonate, sodium bicarbonate, periodontal therapy, instrumentation

## Abstract

Background: Supragingival polishing is a crucial part of nonsurgical periodontal therapy. In recent years, air polishing has been used for this purpose, introducing different polishing powders. The purpose of the following study was to investigate the damage to the gingival margin during air polishing by an inexperienced operator. Methods: Five porcine models were polished by means of three different polishing powders: calcium carbonate, sodium bicarbonate, and erythritol. Their impact on the gingival margin was examined by means of histological and scanning electron microscopical observations and compared to healthy samples and samples polished with a polishing brush and paste. Results: The histological observations revealed superficial to minor lesions limited in the epithelium by all the groups tested. Both examination protocols demonstrated less invasiveness of the erythritol-based polishing powder. Conclusions: Within the limitations of the present study, it could be concluded that air polishing is a safe instrumentation method for periodontal therapy even in inexperienced hands when the exact protocol is followed. The erythritol-based polishing powder seems to provide less of an impact on the gingival margin.

## 1. Introduction

Periodontal and peri-implant health is a fundamental aspect of dental care. There is clear evidence for the bacterial etiology of periodontal diseases. Bacterial biofilm formation initiates gingival inflammation. The etiopathogenesis of periodontitis and peri-implantitis is much more complex and multifactorial, influenced by the host immune response, genetic factors, systemic diseases, smoking, etc. However, studies have confirmed the pivotal role of plaque accumulation since the absence of periodontal pathogens has been shown to be consistent with periodontal health. Therefore, dental biofilm removal is crucial to prevent the onset or recurrence of periodontal diseases. Plaque control consists of personal (daily home) plaque control combined with frequent professional biofilm and calculus removal. The latter includes procedures such as hand and/or ultrasonic instrumentation and teeth polishing. Different instrumentation modalities have been proposed for teeth polishing, including the conventional method with rubber cups, brushes, and polishing paste [1] as well as various air-powder polishing systems, but none has been proven to be superior to the others [2]. Factors such as level of biofilm/staining removal, patient comfort, and procedure duration are considered when evaluating the effectiveness of the different modalities. However, it should be emphasized that all polishing methods are associated with some degree of mechanical trauma to the surrounding tissues. Therefore, effects on tooth surfaces and the gingival margin are the most important variables in evaluating the efficacy of the different modalities. Most of the studies have focused on the adverse effects that polishing can cause on root cementum and restorative materials such as composite resins, cements, and amalgam. In this regard, it has been stated that the aggressive use of rubber cups or bristle brushes may remove the layer of cementum, which is thin in the cervical area [3]. Additionally, those air-powder polishing devices using sodium bicarbonate may cause significant loss of tooth substance. On the other hand, the effects of polishing on the gingiva are somewhat neglected as it is generally accepted that the damage is transient and therefore clinically insignificant. However, there are clinical situations, such as desquamative gingivitis, where the gingiva is thin, painful, and friable and any additional trauma should be avoided. Desquamative gingivitis is a local manifestation of the gingiva regarding some mucocutaneous diseases such as pemphigus vulgaris, pemphigoid, and lichen planus. These are chronic conditions characterized by long-lasting lesions and frequent relapses. Oral hygiene procedures are painful, and patients avoid flossing and brushing, leading to plaque and calculus deposition and subsequent superimposed inflammation, predisposing to further periodontal destruction. Therefore, the treatment of desquamative gingivitis requires frequent professional removal of biofilm and calculus with the selection of the most painless and atraumatic debridement method possible. The soft tissue damage around the teeth depends on the tools used for this purpose as well as on the operator’s skills [1]. Below, we discuss the currently used instrumentation modalities, with an emphasis on their soft tissue effects.

The air polishing devices are mainly used for teeth polishing and dental staining removal. These devices use a slurry of pressurized air, abrasive powder, and water to remove dental biofilm and stains [4]. They seem to provide better effectiveness in biofilm removal in comparison to conventional teeth polishing [5]. Moreover, performing dental prophylaxis by means of air polishing devices seems to be more comfortable and a preferred method of instrumentation not only for clinicians but also for patients [5,6]. According to different authors, air polishing causes not only less discomfort for patients but is less time-consuming and demonstrates a reduced risk of damage to dental restorations compared to the traditional methods as well [7,8]. 

Various materials have been proposed for air polishing: sodium bicarbonate, calcium carbonate, and glycine [4,9]. Each of them demonstrates different properties like abrasiveness that affect the aspect of their clinical application. Some of the earliest powders applied for air polishing are sodium bicarbonate and calcium carbonate with different particle sizes of up to 250 µm. They have been demonstrated to be effective in supragingival biofilm removal, but other types of polishing powders with smaller particles like glycine seem to be safer regarding human root surfaces, gingiva, and dental restorative materials [10,11] Moreover, glycine-based air polishing demonstrates effectiveness in nonsurgical perimucositis and periimplantitis treatment [12]. In recent years, erythritol-based polishing powders have gained scientific interest because of their smaller particles and reduced side effects to the oral hard- and soft tissues [13,14]. Erythritol is a natural sugar alcohol that is approved as a sugar substitute but has also demonstrated excellent biocompatibility [15,16]. Regarding the literature, patients report better acceptance rates and reduced pain levels with erythritol polishing in comparison to the conventional biofilm removal procedures with hand and ultrasonic instruments [17,18]. Erythritol-based polishing powder demonstrates effectiveness regarding active periodontal therapy combined with ultrasonic instrumentation and in the supportive periodontal therapy by reducing probing pocket depth, bleeding on probing, and gain of clinical attachment [17]. One of the newest proposed dental applications of erythritol-based polishing powders is during the supportive periodontal therapy of patients with autoimmune mucocutaneous diseases like oral lichen planus, pemphigus vulgaris, and mucous membrane pemphigoid [18], although some clinical studies report that the application of high pressure and water could be overestimated by some patients [19]. 

The aim of the present ex vivo study was to evaluate the effects of different polishing powders and conventional polishing with a brush and paste on the gingival margin when undergraduate dental students provide this treatment.

## 2. Materials and Methods

Five porcine mandibles with the presence of comparable external supragingival staining and stored no more than 24 h at constant temperature of 4 degrees were obtained from the nearby abattoir. After reaching room temperature, the study was initiated. One mandible was used for negative control and the other four were randomly divided into four groups. Eight teeth from the mandible (premolars and molars) were polished in different approaches. In three groups, polishing devices were used with the relevant powders. In group A, calcium carbonate powder was used with size of the particles 54 µm (Flash Pearl, Nakanishi Inc., Tochigi, Japan) and air polishing devices (Prophy-Mate Neo, NSK, Tokyo, Japan). In group B, sodium bicarbonate powder was used with size of the particles 65 to 40 µm (Classic prophylaxis powder, EMS, Nyon, Switzerland) with air polishing device AIRFLOW^®^ Prophylaxis Master, EMS, Switzerland. In group C, erythritol powder was used with size of the particles 14 µm (Plus prophylaxis powder, EMS, Switzerland) and air polishing device AIRFLOW^®^ Prophylaxis Master, EMS, Switzerland. In the fourth group, group D, polishing brush and paste with calcium carbonate and pumice were used (Clean Polish, Kerr, Brea, CA, USA). Instrumentation finished when clean surfaces were achieved. 

Instrumentation was performed by four students of Faculty of Dental Medicine, Medical University of Plovdiv—fifth year of dental education. They attended the same training program of Periodontology and were included after providing their consent for participation in the study. Students were trained for conducting the polishing methods and were randomly assigned to groups A to D. 

Teeth assigned to air polishing were instrumented only supragingivally with 45 to 90° angulation against the occlusal surface of the teeth until clean surface was reached. 

**Figure 1:**
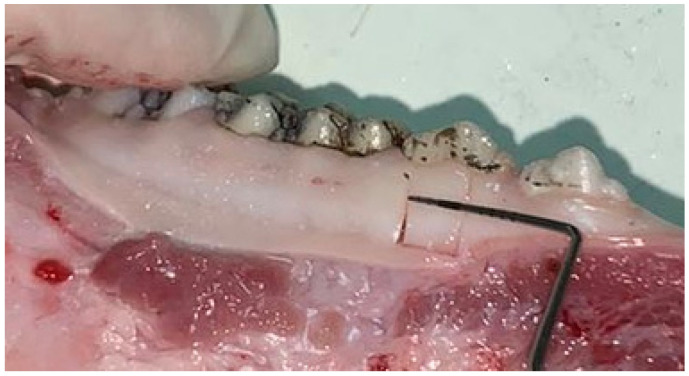
Cadaver biopsy sampling from negative control group, swine lower jaw.

To assess the gingiva damage, excisional biopsies were taken, including gingival margin and part of the attached gingiva (Figure 1). All the biopsies were 5 mm wide and 10 mm long. The biopsy was conducted with one intrasulcular incision, two vertical incisions, and one horizontal incision. Elevator was used from apically to coronally to separate the biopsy without touching the gingival margin. Eight samples were taken from every pig jaw (one from every tooth cleaned). The biopsies were stored in 10% formalin until half of them were used for histological analysis and half for scanning electron microscopy analysis, which were subsequently performed.

For histological evaluation, 4 samples from every mandible were stained with hematoxylin and eosin (H&E) and observed by light microscopy with a microscope equipped with a digital camera (Leica DMC 2900, Leica Microsystems, Danaher, Washington, DC, USA) and software (Leica Application Suite, Version 4.8.0). Tissue samples were assessed based on histological scores outlined in Table 1, modified from Petersilie et al. [19].

For the scanning microscopy observation, one tissue sample from every mandible was enrolled in the study as well as samples from the powders used. All the specimens were prepared with ethanol dehydration and gold sputtering and observed with scanning electron microscope JEOL-JSM- 6390 (Tokyo, Japan). 

## 3. Results

- Histological assessments

**Figure 2:**
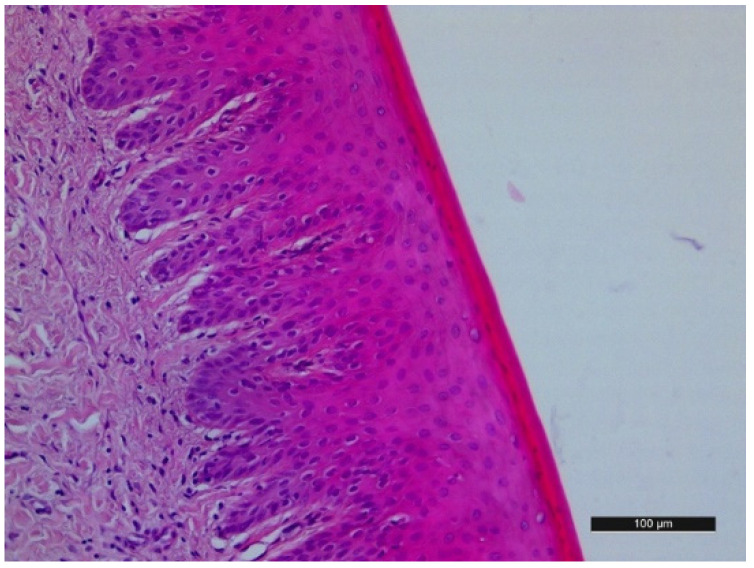
Histological examination of gingival margin around nonpolished teeth—negative control. E—epithelium; BL—basal lamina; CT—connective tissue.

The negative control, untreated gingiva, presented a normal histological structure with an undamaged epithelial layer and well-formed rete pegs with mild lymphocytic infiltration in the submucosal connective tissue (Figure 2).

All the tested groups demonstrated only epithelium damage without reaching the underlying connective tissue. In some of the histological samples, we observed inflammatory changes in the connective tissue due to possible premortem trauma and inflammation in the gingival tissues.

**Figure 3:**
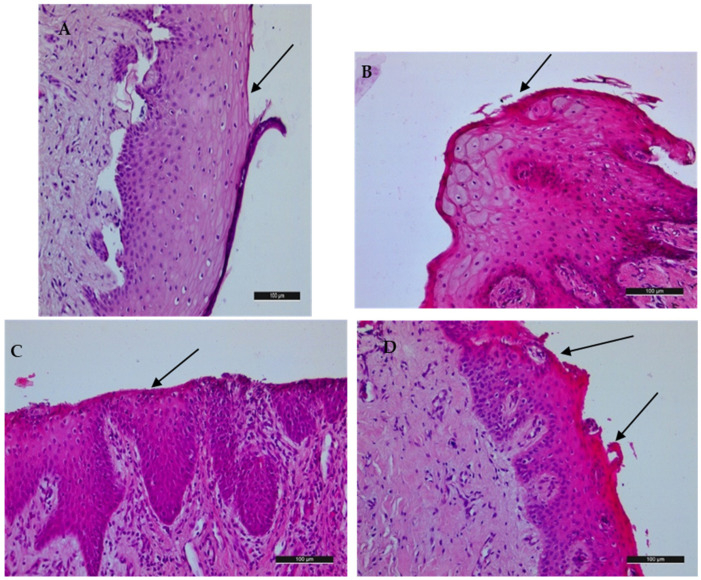
Histological examination of test groups. (**A**) Group A, calcium carbonate powder, 54 µm. (**B**) Group B, sodium bicarbonate powder, 60 to 45 µm. (**C**) Group C, erythritol powder, 14 µm. (**D**) Group D, polishing brush and paste (positive control). Black arrows demonstrate the changes observed in the epithelial layer.

In group A, calcium carbonate polishing powder, degenerative changes in the epithelium (vacuolar degeneration) without any specific changes in the submucosal connective tissue were observed. The changes correspond to a score of 2 pertaining to the scale used, as presented in Table 1 (Figure 3A). There is obvious detachment of the epithelium from the connective tissue, which is a result of the postmortem changes in the gingival tissues. 

In group B, weak desquamation in the superficial epidermal layers was observed, as well as mild cellular edema in the spinous and basal layers and no obvious changes in the connective tissue. The changes correspond to score 2 of the scale used, as indicated in Table 1 (Figure 3B). 

In the group treated with the erythritol-based polishing powder, mild edema was observed in the mucosa and minimal desquamation of the epithelium, without changes in the submucosal connective tissue, corresponding to a score of 1, as indicated in Table 1 (Figure 3C).

In group D, polishing brush and paste, the deepest lesions of the epithelium were observed, although no damage in the connective tissue was demonstrated (score 2) (Figure 3D).

- SEM observation

Scanning electron microscopy revealed various distributions of epithelium damage after polishing. 

**Figure 4:**
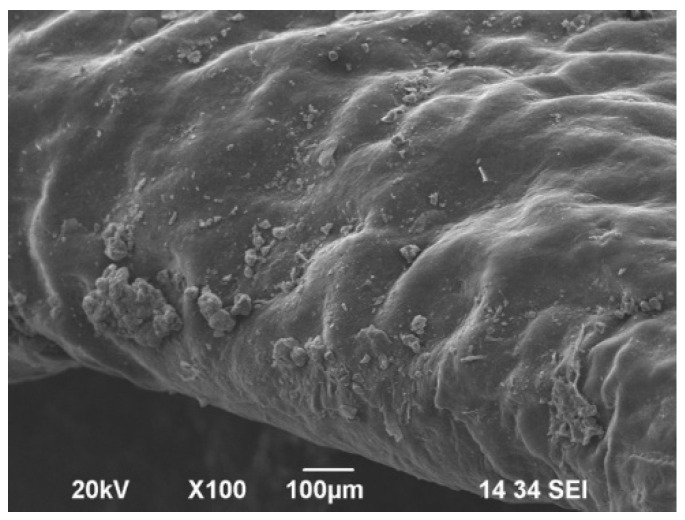
SEM examination of gingival margin around nonpolished teeth—negative control. Magnification ×100.

**Figure 5:**
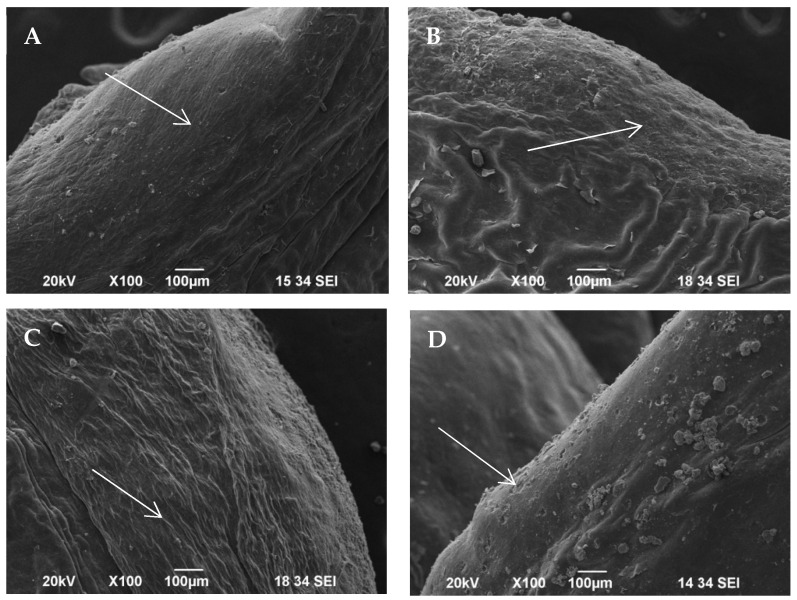
Scanning electron microscopic examination of test groups. (**A**) Group A, calcium carbonate powder, 54 µm. (**B**) Group B, sodium bicarbonate powder, 60 to 45 µm. (**C**) Group C, erythritol powder, 14 µm. (**D**) Group D, polishing brush and paste (positive control). White arrows demonstrate the changes in the epithelium.

The untreated gingiva demonstrated an intact keratinized surface (Figure 4). Some external pollutants could be observed. Figure 5A,B demonstrate an intact epithelium with some remnants from the polishing powders. Overall, the surface appears to be smooth, with limited areas with surface alteration (white arrows). In Figure 5C, the surface appears to be smooth, with no damage to the epithelium. The sample treated with a polishing brush and paste demonstrates the most damage to the epithelium, presenting several areas with lesions (Figure 5D, arrows).

## 4. Discussion

Supragingival polishing is performed with the nozzle directed from 45 to 90 degrees against the enamel of the teeth, aiming at the removal of dental biofilm and extrinsic staining of the enamel. Different polishing powders with particle sizes from 14 to 250 µm have been proposed for this procedure. During this application, some particles of the powder might affect the gingival margin and may cause surface damage due to the high pressure from the polishing system. Although most studies demonstrate no to minimal soft tissue injury during air polishing with different polishing powders [17], there are clinical situations where soft tissue damage during supportive periodontal therapy is an extremely important factor. Mucocutaneous autoimmune diseases like oral lichen planus, pemphigus vulgaris, and pemphigoid are associated with desquamation and erosion regarding the attached gingiva that often spread to the free gingival margin, causing impaired personal and professional oral hygiene [18]. 

In this ex vivo study, three different polishing powders were tested in comparison to conventional prophylaxis instrumentation (positive control). The operators were inexperienced students—fifth year of dental education, trained for the purpose of the study. It was found that different polishing instruments and powders led to different degrees of epithelium damage on the gingival margin, although no severe lesions were found. 

Cytoplasmic vacuolization and intercellular edema in the deep layers of the spinous epithelium as well as the separation of the basal layer from the connective tissue found in our samples are expressions of early postmortem changes like those described by Pradeep et al., 2009 and Patra et al., 2021 [20,21]. Our observations of cellular edema in the gingival spinous epithelium of slaughtered pigs by 18–20 h are comparable to early postmortem changes in hypoxic individuals like those reported by Mochan et al., 2016 [22]. The condition of the epithelium and subcutaneous tissue also corresponds to the changes in the early postmortem period described by Mazzotti et al., 2019 [23]. Regarding the superficial layers of the stratum corneum, their desquamation occurs postmortem much later according to the sources indicated below. Conversely, cadavers, which they used in these studies, were kept in cool rooms or at room temperature (from 3–4 °C to 20–25 °C) to a fraction of that in positive cold rooms in a slaughterhouse (from 0–1 °C to 4–10 °C) and for an interval corresponding to less than 18 to 20 h following slaughter. The changes we observed are also comparable to the postmortem changes found by Mahalakshmi et al., 2016 [24] in gingival mucosa fixed at an interval of 15 min to 4 h after various dental procedures, as well as the early postmortem changes observed up to 4 h by Srirangarajan et al., 2021 [25]. In our opinion, this makes the changes we observed credible, especially given the absence of similar changes, or only minimal ones, in the control group and the groups using gentler polishing materials.

In all the samples, the lesions were limited to the surface epithelium without reaching the connective tissue. The study by Weusmann et al. reported that the angulation of the pressurized powder and water has a major impact on the gingival damage, suggesting that 30–60 degree angulation of the nozzle against the gingiva causes deep gingiva alteration reaching the underlying connective tissue, bearing in mind that they used the polishing powders only against the attached gingiva [26]. In our study, we examined the accidental damage to the free gingival margin during supragingival polishing of teeth surfaces, which resembles the procedure of a real clinical situation. We determined that gingival samples instrumented with a polishing brush and paste demonstrated the deepest alteration to the gingiva according to the histological examination (Figure 3D) and mostly widespread disturbances according to the SEM examination (Figure 5D). This could be due to the presence of pumice in the polishing paste we used. In contrast, those surfaces treated with the erythritol-based polishing powder demonstrated superficial damage to the gingival samples, which was proven by histological and scanning electromicroscopy observations. The calcium carbonate and sodium bicarbonate polishing powders, which were composed of particles of a similar size between 40 and 65 µm, demonstrated comparable surface damage regarding the gingival samples, involving minor lesions in the epithelial layers limited to small areas. These results are comparable to the results achieved by Petersilka et al., who demonstrated that glycine polishing powder and sodium bicarbonate polishing powder are less invasive than hand instrumentation [17]. We could not find other studies examining soft tissue damage by erythritol-based polishing powders. But, we demonstrated that the particles of this powder that were the smallest particles according to the manufacturer—14 µm—produced only superficial alterations regarding the gingival margin that are limited to small areas. This is extremely important, especially in cases with mucocutaneous conditions of the gingiva where soft tissue damage during supportive periodontal therapy is of paramount importance. Regarding some of the mucocutaneous diseases like lichen planus, the so-called Koebner phenomenon was described, which involves the appearance of new skin lesions after trauma [27]. Thus, it could be speculated that traumatic professional periodontal maintenance could lead to new oral mucosa lesions, which implies the necessity of careful and atraumatic instrumentation. 

Air abrasion polishing devices have proven their efficacy in biofilm removal from the enamel and cementum surfaces without causing damage to the teeth or composite restorations and implant surfaces [4,7,12]. A limited number of studies have tested the soft tissue response to air abrasion polishing devices [19,26,28]. Most of them demonstrated no serious damage to the gingival margin and emphasize that air polishing could lead to local trauma correlated to instrumentation time and the principles and angulation of the air polishing procedure. The topic of soft tissue damage during supportive periodontal therapy is extremely important for patients with mucocutaneous diseases, where personal oral hygiene is limited and improper professional oral hygiene could lead to further lesions on the oral mucosa. Finding a less invasive supportive periodontal therapy for such patients is of paramount importance, but more histological and clinical studies are needed to prove the efficacy and safety of air polishing regarding the soft tissues. The current study is limited in its design, and factors such as the duration of the polishing process, distance of the nozzle tip to the tooth surface, spray pattern, and pressure were not taken into consideration. Further laboratory and clinical studies could improve the accuracy of the findings.

## 5. Conclusions

Our pilot study demonstrates that all the tested polishing methods, including air polishing powders and traditional polishing brushes, caused damage limited to the superficial epithelial layers without reaching the connective tissue of the gingival margin. The results from the present study demonstrate that the air polishing devices are safe modalities for teeth polishing even when it is conducted by an inexperienced operator. They could be used efficiently in supportive periodontal and peri-implant therapy without causing soft tissue damage. In contrast, the traditional polishing brushes and pastes demonstrated certain damage to the superficial epithelium layers, presenting limitations of this pilot study. The calcium-carbonate-based and sodium-bicarbonate-based air polishing powders exhibited minor damage in limited areas of the superficial epithelium. The interpretation of the results suggests that erythritol-based air polishing powder demonstrates the most insignificant damage in the epithelium. Extending the present study and conducting more detailed experiments could provide better information regarding the efficacy and safety of the polishing methods and devices. 

## Figures and Tables

**Table 1:** Histological scoring for evaluation of gingival damage.

Score	Histological Observation
0	No lesion, undamaged epithelium, and connective tissue
1	Superficial lesion, disruption of superficial epithelial layers
2	Minor lesion, disruption of superficial epithelial layers, no connective tissue damage
3	Medium lesion, Superficial layers of the epithelium removed, basal membrane partially damaged
4	Severe lesion, epithelium and basal membrane completely removed, connective tissue exposed

## Data Availability

The original contributions presented in the study are included in the article, further inquiries can be directed to the corresponding author.
